# Duodenal ileus caused by a calf feeding nipple in a cow

**DOI:** 10.1186/1746-6148-7-2

**Published:** 2011-01-06

**Authors:** Ueli Braun, Charlotte Schnetzler, Matteo Previtali, Christian Gerspach, Tanja Schmid

**Affiliations:** 1 Department of Farm Animals, University of Zurich, Winterthurerstrasse 260, 8057 Zurich, Switzerland

## Abstract

**Background:**

The aim of this report was to describe duodenal obstruction caused by a rubber foreign body in a cow.

**Case Presentation:**

The clinical, biochemical and ultrasonographic findings in a five-year-old Swiss Braunvieh cow with duodenal ileus caused by a calf feeding nipple are described. The main clinical signs were anorexia, ruminal tympany, decreased faecal output and abomasal reflux syndrome. Ultrasonographic examination revealed reticular hyperactivity and a dilated duodenum. A diagnosis of duodenal ileus was made and the cow underwent right-flank laparotomy, which revealed a dilation of the cranial part of the duodenum because of obstruction by a pliable foreign body. This was identified via enterotomy as a calf feeding nipple. The cow was healthy at the time of discharge four days after surgery and went on to complete a successful lactation.

**Conclusions:**

To our knowledge, this is the first description of duodenal obstruction by a calf feeding nipple. This is an interesting case, which broadens the spectrum of the causes of duodenal ileus, which is usually caused by obstruction of the duodenum by a phytobezoar.

## Background

Ileus of the duodenum results in abomasal reflux syndrome, which is characterised by rapid deterioration of the condition and demeanour in cattle [1]. Phytobezoars are the most common cause of obstruction of the duodenum. They are formed when fibrinous adhesions involving the reticulum impair the mechanism responsible for sorting the ingesta, which allows poorly digested feed to move into the abomasum, omasum and duodenum, where it may cause an obstruction of the duodenum [1,2]. In rare instances, coagulated blood may obstruct the duodenum [1,3-5], although this happens more commonly in the jejunum [5]. Trichobezoars may cause duodenal obstruction in calves [6]. Other causes in mature cattle are obstruction of the duodenum by gravel [7], mechanical compression of the duodenum by an abscess in the liver or omentum, or lymphosarcoma [3]. Functional stenosis of the duodenum refers to ileus in which a mechanical cause cannot be identified [8]. The duodenum may also be obstructed by pressure from the gravid uterus [9]. The aim of this report was to describe duodenal obstruction caused by a rubber foreign body.

## Case presentation

A five-year-old Swiss Braunvieh cow was referred to the Department for Farm Animals, University of Zurich, because of a four-day history of anorexia and recent ruminal tympany, which were unresponsive to various medical treatments. The cow was nine-weeks pregnant and had a moderately abnormal general condition. The eyes were sunken and there was reduced skin turgor, a cold muzzle and injected scleral vessels. The heart rate was 68 beats per minute, the respiratory rate 24 breaths per min and the rectal temperature 38.6°C. The abdomen was markedly distended. On rectal examination, an L-shaped distended rumen was palpated and there were small amounts of dark faeces that contained mucus. Ruminal contractions were reduced and normal layering of the contents was absent at palpation. Swinging and percussion auscultation of the abdomen were negative. The abdominal wall was tense and intestinal sounds were almost completely absent. Of the foreign body tests, the withers pinch was positive and percussion of the reticulum and pole test were negative.

The results of macroscopic examination of a urine sample and a urine test strip were normal. The haematocrit was increased at 45% (normal, 30 - 35%) and the concentration of total solids was 104 g/l (normal, 60 - 80 g/l). Urea and creatinine concentrations were increased at 18.6 mmol/l (normal, 2.4 - 6.5 mmol/l) and 320 μmol/l (normal, 55 - 103 μmol/l), respectively, and chloride, potassium and calcium concentrations were decreased at 66 mmol/l (normal, 100 - 105 mmol/l), 3.1 mmol/l (normal, 4.0 - 5.0 mmol/l) and 1.9 mmol/l (normal, 2.3 - 2.5 mml/l), respectively. Inorganic phosphorus was increased at 4.6 mmol/l (normal, 1.3 - 2.4 mmol/l). The chloride concentration of ruminal juice was markedly increased at 78 mmol/l (normal, 15 - 30 mmol/l). Blood gas analysis revealed compensated metabolic alkalosis with a base deficit of +20.3 mmol/l (normal, -2 to +2 mmol/l), a bicarbonate concentration of 43.9 mmol/l (normal 20 - 30 mmol/l) and a pH of 7.45 (normal, 7.40 - 7.50).

**Figure 1:**
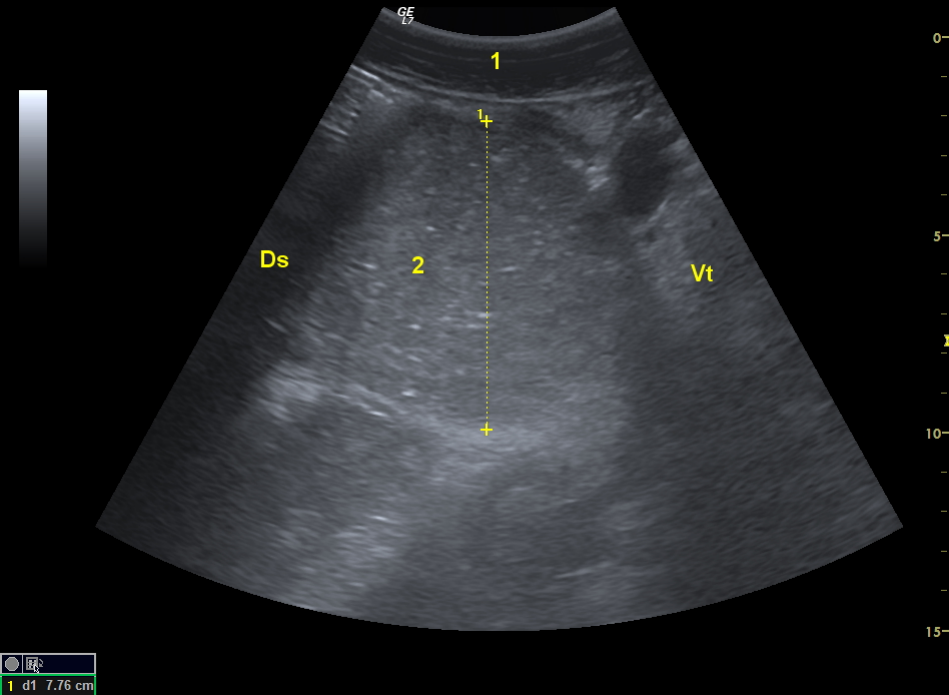
**Ultrasonogram of the duodenum**. Ultrasonogram of the cranial part of the duodenum of a cow with obstruction of the duodenum by a rubber calf feeding nipple. The duodenum is severely dilated and has a diameter of 8 cm. 1 Abdominal wall, 2 Duodenum, Dotted line Diameter of the duodenum, Ds Dorsal, Vt Ventral.

On ultrasonography, the reticulum had six biphasic contractions per 3 minutes (normal, 3 - 4 contractions/3 minutes) [10] and its contour was normal. The rumen, omasum and abomasum were severely dilated; the rumen was in close proximity to the right abdominal wall and the abomasum extended to a point 10 cm cranial to the right stifle. The cranial part of the duodenum had no contractions and was dilated with a diameter of 7.9 cm (Figure 1). The remainder of the intestinal tract was empty and atonic.

**Figure 2:**
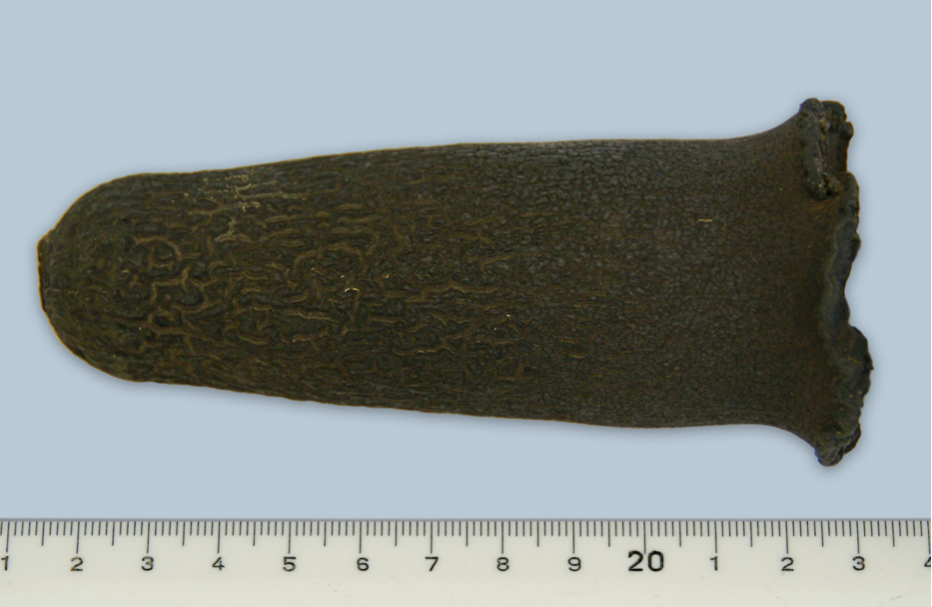
**Calf feeding nipple**. Calf feeding nipple after removal from the duodenum.

Based on all the findings, a diagnosis of duodenal ileus was made. A standing right-flank laparotomy was carried out under proximal paravertebral anaesthesia. A 25-cm incision was made 5 cm caudal and parallel to the last rib, and the abdominal cavity was explored. The main findings were severely dilated forestomachs and abomasum, and a pliable foreign body in the sigmoid flexure of the duodenum, which caused dilation of the duodenum proximally. Distal to the obstructing foreign body, the intestines were empty and had no contractions. The foreign body was massaged in a retrograde direction to a position just distal to the pylorus, and that part of the duodenum was exteriorised. Clamps were placed on the duodenum proximal and distal to the foreign body, which was then removed via enterotomy, and identified as a rubber calf feeding nipple (Figure 2). The enterotomy incision was closed in two layers (simple continuous followed by Cushing suture) using monofilament absorbable suture material (USP 2/0, Biosyn, Virbac, Küsnacht) and rinsed liberally with isotonic saline solution. The peritoneum, fascia and transverse abdominal muscle were sutured using USP 2 absorbable braided suture material (Polysorb™, Syneture, USA) in a simple continuous suture pattern. The internal and external oblique abdominal muscles (USP 2) and subcutaneous tissue (USP 0) were adapted and closed separately using absorbable suture (Polysorb, Syneture, Norwalk, Connecticut) in a continuous suture pattern. The skin was closed with skin staples (Auto Suture, Tyco Healthcare Group LP, Norwalk, Connecticut). Postoperatively, the cow received 9 × 10^6 ^IU of procaine penicillin (Procacillin, Intervet, Zurich), intramuscularly, three times daily for 3 days; 500 mg of flunixin meglumine (Fluniximin, Graeub, Berne), intravenously, once daily for 3 days, and 10 litres of sodium chloride and glucose solution (50 g glucose and 9 g NaCl/litre) daily via an indwelling intravenous catheter for 3 days. In addition, the cow received 500 ml of a 40% calcium borogluconate solution (Calcamyl-40 MP, Graeub, Bern) immediately after surgery and 5 litres of sodium chloride and glucose solution containing 42.5 mg neostigmine (Konstigmin, Vétoquinol, Ittigen) administered at a flow rate of 0.0015 mg neostigmine/kg/hour during the following 48 hours via the indwelling catheter.

One day after surgery, the demeanour of the cow had improved, and she started eating small amounts of feed and passing olive-coloured faeces with a soft pulpy consistency. The general condition, appetite and faecal output were normal on the following day. The cow was discharged four days postoperatively and the skin staples were removed ten days after the surgery. A follow-up via telephone seven months later revealed that the cow had recovered completely and produced more than 10,000 kg of milk during the current lactation.

The clinical signs of the cow described in this report were typical of duodenal ileus [1,2]. They included rapid deterioration of the general condition, an enlarged L-shaped rumen and markedly diminished faecal output, but no signs of colic and an absence of dilated intestinal loops on transrectal palpation. The striking ruminal findings can be misinterpreted as an underlying ruminal problem rather than an intestinal disorder. Laboratory findings indicating abomasal reflux are typically seen with impaired passage of ingesta in the abomasum or proximal small intestine and have been described in cows with duodenal ileus of varying causes [1,3,4]. Similar laboratory findings are also seen in cows with abomasal displacement and pyloric stenosis [11]. Haemoconcentration, azotaemia, hypochloraemia and hypokalaemia are considered to be the main cause of rapid deterioration in the general condition of cattle with duodenal ileus.

Ultrasonography allowed localisation of the ileus to the duodenum and differentiation of the problem from pyloric and jenunal stenosis. With the former, the abomasum is dilated and the small intestines are empty, whereas with the latter multiple dilated loops of small intestine with a diameter of at least 4 cm are typically seen [12]. One to three dilated intestinal loops in the cranial abdomen are typical of duodenal ileus [13,14]. The normal diameter of the duodenum is 0.9 to 5.5 cm [15], whereas in cows with duodenal ileus, diameters may range from 6.5 to 9.9 cm [13]. Of special interest were the reticular findings because six biphasic contractions per three minutes indicated reticular hypercontractility. Similar findings have been described in cows with reticulo-omasal stenosis [16] and vagal indigestion [17].

Treatment of duodenal ileus consists of surgical removal of the inciting cause via right-flank laparotomy, combined with the administration of sodium chloride, glucose, calcium, potassium, non-steroidal antiinflammatory drugs and antibiotics [1,3]. In uncomplicated cases, the obstructing duodenal content may be manually disintegrated and moved distally. However, this is not usually feasible and an enterotomy is required in most cases. In the present case, a rubber foreign body, rather than a bezoar or a blood clot, was immediately suspected after palpation of the duodenum. It would be interesting to know whether the nipple was ingested during the nursing period or whether it was accidentally eaten by the cow later.

## Conclusion

Duodenal ileus is usually caused by obstruction of the duodenum by a phytobezoar. In rare instances, coagulated blood may obstruct the duodenum or the duodenum is mechanically compressed by an abscess or a neoplasia. To our knowledge, this is the first description of duodenal obstruction by a calf feeding nipple. This is an interesting case, which broadens the spectrum of the causes of duodenal ileus.

## Competing interests

The authors declare that they have no competing interests.

## Authors' contributions

UB supervised the clinical and ultrasonographic examination, reviewed the literature and prepared the manuscript. CS and MP carried out the clinical examination. CG performed the ultrasonographic examination. TS performed the laparotomy. All authors read and approved the final manuscript.

## References

[B1] Braun U Steiner A Götz M Clinical signs, diagnosis and treatment of duodenal ileus in cattle Schweiz Arch Tierheilk 1993 135 345 355 8266052

[B2] Garry F Hull BL Rings DM Hoffsis G Comparison of naturally occurring proximal duodenal obstruction and abomasal volvulus in dairy cattle Vet Surg 1988 17 226 233 10.1111/j.1532-950X.1988.tb01002.x 3238895

[B3] Anderson DE Ivany Ewoldt JM Intestinal surgery of adult cattle Vet Clin North Am (Food Anim Pract) 2005 21 133 154 10.1016/j.cvfa.2004.12.010 15718090

[B4] Lejeune B Lorenz I Ultrasonographic findings in 2 cows with duodenal obstruction Can Vet J 2008 49 386 388 18481548 PMC2275343

[B5] Braun U Forster E Steininger K Irmer M Gautschi A Previtali M Gerspach C Nuss K Ultrasonographic findings in 63 cows with haemorrhagic bowel syndrome Vet Rec 2010 166 79 81 10.1136/vr.c178 20081179

[B6] Anderson DE Surgical diseases of the small intestine Vet Clin North Am (Food Anim Pract) 2008 24 383 401 10.1016/j.cvfa.2008.02.004 18471577

[B7] Cebra CK Garry FB Gravel obstruction in the abomasum or duodenum of two cows J Am Vet Med Assoc 209 1294 1296 8837655

[B8] Van der Velden MA Functional stenosis of the sigmoid curve of the duodenum in cattle Vet Rec 1983 112 452 453 10.1136/vr.112.19.452 6868314

[B9] Koller U Lischer C Geyer H Dressel C Braun U Strangulation of the duodenum by the uterus during late pregnancy in two cows Vet J 2001 162 33 37 10.1053/tvjl.2000.0543 11409927

[B10] Braun U Rauch S Ultrasonographic evaluation of reticular motility during rest, eating, rumination and stress in 30 healthy cows Vet Rec 2008 163 571 574 10.1136/vr.163.19.571 18997187

[B11] Radostits OM Gay CC Hinchcliff KW Constable PD Diseases of the alimentary tract - II Veterinary Medicine. A Textbook of the Diseases of Cattle, Horses, Sheep, Pigs, and Goats 2007 Philadelphia, Saunders Elsevier 293 382

[B12] Braun U Ultrasonography in gastrointestinal disease in cattle Vet J 2003 166 112 124 10.1016/S1090-0233(02)00301-5 12902177

[B13] Braun U Marmier O Pusterla N Ultrasonographic examination of the small intestine of cows with ileus of the duodenum, jejunum or ileum Vet Rec 1995 137 209 215 10.1136/vr.137.9.209 7502471

[B14] Braun U Ultrasonography of the gastrointestinal tract in cattle Vet Clin North Am (Food Anim Pract) 2009 25 567 590 10.1016/j.cvfa.2009.07.004 19825434

[B15] Braun U Marmier O Ultrasonographic examination of the small intestine of cows Vet Rec 1995 136 239 244 10.1136/vr.136.10.239 7785178

[B16] Braun U Schweizer G Flückiger M Radiographic and ultrasonogra-phic findings in three cows with reticulo-omasal obstruction due to a foreign body Vet Rec 2002 150 580 581 10.1136/vr.150.18.580 12019653

[B17] Braun U Rauch S Hässig M Ultrasonographic evaluation of reticular motility in 144 cattle with vagal indigestion Vet Rec 2009 164 11 13 10.1136/vr.164.1.11 19122215

